# Clinical Impacts and Risk Factors for Central Line-Associated Bloodstream Infection: A Systematic Review

**DOI:** 10.7759/cureus.40954

**Published:** 2023-06-25

**Authors:** Khadejah M Alshahrani, Afnan Z Alhuwaishel, Norah M Alangari, Malak A Asiri, Norah A Al-Shahrani, Ahmed A Alasmari, Osama J Alzahrani, Abdulaziz Y Ayedh, Meshari M Qitmah

**Affiliations:** 1 Department of Internal Medicine, Aseer Central Hospital, Abha, SAU; 2 Department of Internal Medicine, King Fahad Medical City, Riyadh, SAU; 3 Department of Internal Medicine, Armed Forces Hospital Southern Region, Khamis Mushait, SAU; 4 Department of Internal Medicine, King Fahad Specialized Hospital, Tabouk, SAU

**Keywords:** morbidity, predictors, risk factors, clabsi, central line-associated bloodstream infection

## Abstract

Background

A central line-associated bloodstream infection (CLABSI) is defined as a primary bloodstream infection (BSI) in a patient that had a central line within the 48-hour period before the development of the BSI and is not bloodstream-related to an infection at another site. CLABSI is a common healthcare-associated infection and a significant cause of morbidity and mortality.

Methods

This systematic review included studies published within the past 13 years that examined risk factors and clinical impact variables associated with CLABSI, using the Centers for Disease Control (CDC)/National Healthcare Safety Network (NHSN) criteria for defining catheter-associated infection, and included participants of all ages. The terms “CLABSI,” “central line-associated bloodstream infection,” “risk factors,” “predictors,” “morbidity,” “mortality,” “healthcare costs,” and “length of hospital stay” were used to find relevant publications on PubMed/Medline, Google Scholar, and Science Direct. The quality assessment of the included publications utilized the modified Newcastle-Ottawa scale (NOS) for observational studies.

Results

After the full-text screening, we identified 15 articles that met our inclusion and exclusion criteria. The majority of these studies were of good quality and had a low risk of bias based on our bias assessment. The studies included a total of 32,198 participants and covered a time period from 2010 to 2023. The mean age of the male patients included in the studies ranged from 0.1 months to 69.1 years. All of the included studies were either observational cohort studies, cross sectional studies, case-control studies, or case reports. The major study parameters/outcomes extracted were risk factors, CLABSI-associated mortality, hospital cost, length of hospital stay, and catheter days. With respect to predisposing factors, multilumen access catheters were identified as risk factors in three studies, use of more than one central venous catheter per case in four studies, hematologic malignancy in three studies, catheterization duration in four studies, surgical complexity in four studies, length of ICU stays in three studies, and parenteral nutrition in two studies.

Conclusion

The decision to place a venous device should be carefully considered by evaluating individual risk factors for the development of CLABSI. This is important due to the potential for severe clinical consequences and significant healthcare expenses associated with this complication.

## Introduction and background

A central line-associated bloodstream infection (CLABSI) is a primary bloodstream infection (BSI) in a patient that had a central line within the 48-hour period before the development of the BSI and is not bloodstream-related to an infection at another site [1]. CLABSI is a common healthcare-associated infection and is a significant cause of morbidity and mortality. The incidence of CLABSI varies depending on the setting and population studied, but estimates suggest that the incidence is between 0.5 and 5 per 1,000 catheter days [2]. The development of CLABSI is linked to several risk factors. [3,4]. Prolonged catheterization is a major risk factor, as the longer a patient has a central line in place, the higher the risk of developing CLABSI with odds ratios in various studies ranging from 1.028 to 5.52 [5]. This is because bacteria have more time to colonize the catheter and cause an infection. Immunocompromised states, such as those in cases with weakened immune systems, such as those undergoing chemotherapy, transplant recipients, and those with HIV, are at a higher chance of developing CLABSI [6]. This also includes patients with underlying medical conditions such as cancer, renal failure, or liver disease, which weaken the body’s immune system. Other risk factors include inadequate hand hygiene, poor catheter insertion technique, and the use of uncoated catheters [7]. Catheters that are not coated with antimicrobial substances are more likely to become colonized with bacteria. Central line insertion during emergencies, such as cardiac arrest or trauma, can increase the risk of infection as healthcare providers may not have enough time to properly prepare for the procedure [8]. The risk also increases with the use of multiple catheters, as patients who have more than one catheter in place, or who require frequent catheter changes, are at a higher likelihood of developing CLABSI. The most common microorganisms associated with CLABSI are bacteria, particularly those that are commonly found on the skin or in the environment. These include *Enterobacteriaceae* species (23-31%), *Staphylococcus aureus*, particularly methicillin-resistant *Staphylococcus aureus* (MRSA) (16%), *Candida* species (27.6%), coagulase-negative staphylococci, *Enterococcus* species, and *Pseudomonas aeruginosa* [9-11]. Accurate identification of the microorganism causing the infection is important for appropriate treatment with antibiotics. CLABSI can have a significant clinical impact, including prolonged hospitalization, increased healthcare costs, and increased mortality. Patients with CLABSI are at risk of developing sepsis, organ failure, and other serious complications [12]. In addition, CLABSI can lead to the development of antimicrobial-resistant infections, which can be difficult to treat and increase the risk of adverse outcomes [13]. CLABSI prevention efforts, including proper insertion and meticulous maintenance of central lines, play a vital role in mitigating the occurrence and clinical ramifications of this infection [14]. The objective of this study is to investigate the clinical implications and risk factors of CLABSI, focusing on the role of prevention efforts in mitigating CLABSI occurrence and clinical ramifications.

## Review

Methods

Definition of Outcomes and Inclusion Criteria

The inclusion criteria included studies involving participants aged 0 years or older, addressing risk factors and/or clinical impact variables associated with CLABSI, and utilizing the Centers for Disease Control (CDC)/National Healthcare Safety Network (NHSN) criteria for defining catheter-associated infection. Studies published within the past 13 years were considered, focusing on retrospective or prospective cross-sectional and cohort studies, as well as case reports. Only original articles and English language publications were considered for this systematic review. Abstracts, conference publications, unpublished studies, and case reports or case series with under five cases were excluded. 

Search Strategy

Relevant articles meeting the predetermined eligibility criteria were identified by conducting searches on online databases including PubMed/Medline, Google Scholar, and Science Direct. Electronic searches were enhanced through the use of Boolean operators. The search terms used included “central line-associated bloodstream infection” OR “CLABSI” AND “risk factors,” OR “predictors”; AND “morbidity” OR “mortality'” AND “healthcare costs'” AND “length of hospital stay” OR “duration' to retrieve relevant publications.” Additionally, the reference lists of suitable articles were examined to identify additional relevant publications.

Screening and Extraction

After importing all studies with abstracts and titles into Endnote (version EndNote X8; Clarivate Analytics, London, United Kingdom), duplicate papers were excluded. Subsequently, two investigators independently screened the titles and abstracts based on the predetermined inclusion and exclusion criteria. In the second phase, the full text and abstracts of the remaining papers were carefully examined to determine if they met the inclusion criteria. Two researchers independently assessed the entire texts. Once all relevant articles were identified, a structured extraction sheet was created, focusing on the targeted outcomes. This sheet included information such as study design, country of study, total number of participants, mean age, gender distribution, prevalence/infection rate, risk factors identified, CLABSI-associated mortality, hospital costs, length of hospital admission, and catheter days, which served as baseline outcomes for the analysis.

Quality Assessment

We employed the modified Newcastle-Ottawa scale (NOS) to assess the quality of cross-sectional, cohort, and case-control studies [15-17]. This scale consists of three main domains: methodological quality, comparability, and outcome assessment and reporting. Each category is assigned a maximum of five, two, and three stars, respectively. Factors such as power estimation, sequential participant selection, and potential selection bias were evaluated to assess the study's methodological quality. Comparability was determined by examining whether the study accounted for participant age and other relevant risk factors such as implementation of infection control measures. Studies with minimal risk of bias are typically awarded a maximum of five stars. Based on a scale ranging from 0 to 10, studies were categorized as poor (0-4), satisfactory (5-6), good (7-8), or very good (9-10) in terms of their quality.

Results

Search Results

**Figure 1:**
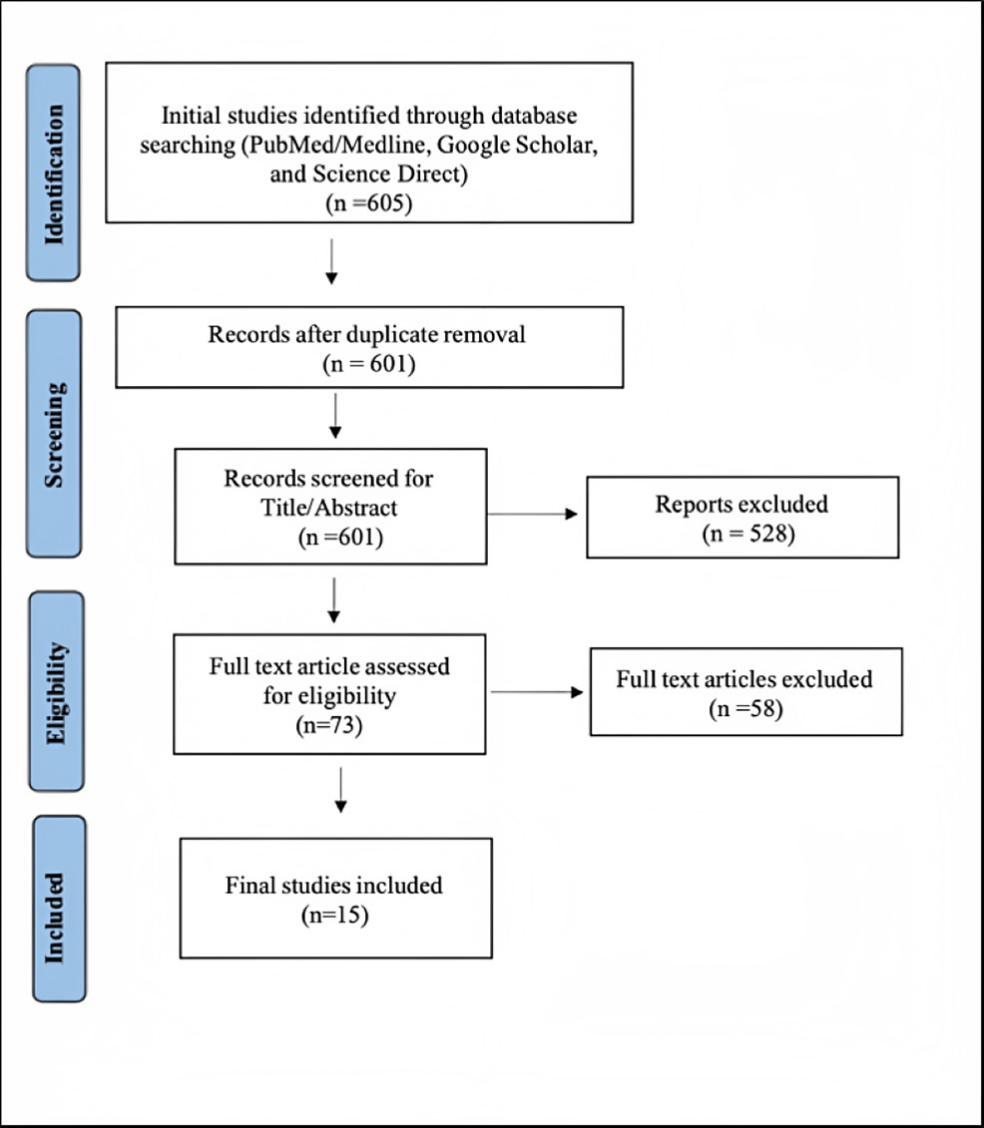
PRISMA flow diagram [33] PRISMA, Preferred Reporting Items for Systematic Reviews and Meta-Analyses

A total of 605 citations were initially identified through the search methods described. After removing duplicates, the number was reduced to 601. Following the screening of titles and abstracts, only 73 citations remained for further consideration. After conducting a full-text screening, only 15 articles met the inclusion and exclusion criteria [18-32]. Abstracts, conference publications, unpublished studies, and case reports or case series with under five cases were excluded as well as studies published in other languages than English. The entire search and screening process is illustrated in Figure 1.

Results of Quality Assessment

The majority of the included studies exhibited good quality and had a low risk of bias, as determined by our bias assessment. Among the studies, eight were classified as good or very good in terms of quality [18,21,24,25,27,30-32]. However, three studies were found to have unsatisfactory quality [20,22,28], as indicated in Tables 1-3).

**Table 1:** Overview of bias assessment results using the modified Newcastle-Ottawa scale for included studies: a cohort study

	Selection				Comparability		Outcome			Total quality score	Quality	
Study	Representativeness of exposed cohort	Selection of nonexposed cohort	Ascertainment of exposure	Demonstration that outcome of interest was not present at the start of the study	Adjust for the most important risk factors	Adjust for other risk factors	Assessment of outcome	Follow-up length	Loss to follow-up rate			
												Advani et al. [18]	1	1	1	0	0	0	2	1	1	7	Good	
Stevens et al. [19]	0	1	1	1	1	0	1	1	0	6	Satisfactory	
Khieosanuk et al. [20]	0	0	0	1	0	0	0	1	0	2	Unsatisfactory	

**Table 2:** Cross-sectional study

		Selection			Comparability	Outcome		Total quality score	Quality
Study	Representativeness of the sample	Sample size	Non-respondents	Ascertainment of the exposure (risk factor)	Confounding factors controlled	Assessment of outcome	Statistical test		
Lissauer et al. [21]	1	0	1	2	2	2	1	9	Very good
Rhee et al. [22]	1	0	0	0	0	1	1	3	Unsatisfactory
Wong et al. [23]	1	0	1	1	1	1	1	6	Satisfactory
Herc et al. [24]	1	0	1	2	2	2	1	9	Very good
Kim et al. [25]	1	0	1	2	1	2	1	8	Good
Torre et al. [26]	0	0	0	1	1	2	1	5	Satisfactory
Malek et al. [27]	0	1	1	2	1	2	1	8	Good
Hernández-Aceituno et al. [28]	0	0	1	0	1	1	1	4	Unsatisfactory
DiPietro et al. [29]	1	0	0	2	1	1	1	6	Satisfactory
Ahn et al. [30]	1	0	1	2	2	1	1	8	Good

**Table 3:** Case-control study

	Selection				Comparability	Exposure			Total quality score	Quality
Author	Adequate case definition	Representativeness of the cases	Selection of Controls	Definition of controls	design or analysis	Assessment of exposure	Same method of ascertainment for cases and controls	Non-response rate		
Baier et al. [31]	1	1	1	1	1	1	1	0	7	Good
Jeong et al. [32]	1	1	1	1	0	1	1	1	7	Good

Study Characteristics of the Included Studies

Finally, a total of 15 studies that met the eligibility criteria were included in this systematic review [18-32]. The studies enrolled participants between 2010 and 2023, including 32,198 patients. The mean age of the included patients ranged between 0.1 months and 69.1 years. The percentage of male participants ranged from 44.1% to 72.3% across eight studies [18-20,25-27,30,31]. All of the included research investigations were observational cohort studies [18-30], case-control studies, or case reports [31,32]. In terms of the countries represented in the included studies, the United States was the focus of six studies [18,19,21,22,24,29], followed by South Korea with three studies [25,30,32]. Australia [23], Brazil [26], Egypt [27], Germany [31], Spain [28], and Thailand [20] were each represented by one study. The key characteristics of the included studies are summarized in Table 4.

**Table 4:** Overview of baseline characteristics in the included studies CLABSI, central line-associated bloodstream infection; GA, gestational age; NR, not reported

Studies	Country	Study design	Year of publication	Sample size	Mean/median age	Gender (male %)
Dipietro et al. [29]	USA	Observational analysis	2020	17,846	NR	NR
Herc et al. [24]	USA	Comparative study	2017	23,088	>65: 11,392; <64: 5,415	NR
Hernández-Aceituno et al. [28]	Spain	Observational analysis	2020	584	G1: 68.0 (12.6); G2: 69.1 (12.4)	NR
Kim et al. [25]	South Korea	Multicenter study	2018	612	Tunneled: 68.0 ± 15.9; conventional: 68.7 ± 14.6	44.4
Lissauer et al. [21]	USA	Comparative study	2012	961	26	NR
Rhee et al. [22]	USA	Observational analysis	2015	104	56.5 ± 14.2 (20–84)	NR
Advani et al. [18]	USA	Comparative study	2010	683	5 (2–8)	56.4
Torre et al. [26]	Brazil	Multicenter study	2018	170	32	57.1
Wong et al. [23]	Australia	Observational analysis	2016	6,353	CLABSI: 54 (36–68); No CLABSI: 61 (46–73)	NR
Stevens et al. [19]	USA	Retrospective cohort study	2013	398	117	62.1
Jeong et al. [32]	South Korea	Case-control study	2021	141	31±5 weeks (GA)	NR
Malek et al. [27]	Egypt	Observational analysis	2018	499	58.2 ± 14.6	72.3
Ahn et al. [30]	South Korea	Observational analysis	2023	2,189	65.0 (54.0–74.0)	58.8
Baier et al. [31]	Germany	Case-control study	2020	610	47	61.5
Khieosanuk et al. [20]	Thailand	cohort study	2021	1,048	0.1 (IQR: 0.03-0.3) months	67.1

Table 5 provides a summary of the main study outcomes, including risk factors, mortality associated with CLABSI, hospital costs, length of hospital stay, and catheter days.

**Table 5:** Outcomes measures of included studies APACHE II, Acute Physiologic and Chronic Health Evaluation version II; CCI, Carlson Comorbidity Index; CLABSI, central line-associated bloodstream infection; CVC, central venous catheter; DRG, diagnosis-related group; HAI, healthcare-associated infection; ICU, intensive care unit; PICC, peripherally inserted central catheters; IR, incidence density rate; SICU, surgical intensive care unit; TPN, total parenteral nutrition; USD, United States Dollars

Study	Prevalence/infection rate	Risk factors	CLABSI-associated mortality	Hospital cost (USD 2,010)	Length of hospital admission	Catheter days
Dipietro et al. [29]	69%	Younger age, greater surgical complexity, and total catheter days	127	NR	NR	58 (28–135)
Herc et al. [24]	1.10%	Hematological cancer, insertion, multilumen PICC, solid cancers with ongoing chemotherapy, CLABSI within 3 months of PICC, receipt of TPN, presence of another CVC at the time of PICC placement	NR	NR	4 (2–8)	NR
Hernández-Aceituno et al. [28]	G1: 5.05%; G2: 2.28%	Replacement of CVC, two or more catheterizations, parenteral nutrition	NR	NR	G1: 20.3 (15.6); G2: 18.3 (14.3)	G1: 6.8 (5.1); G2: 7.7 (6.6)
Kim et al. [25]	NR	CLABSI proved to be significantly less frequent after tunneling than after conventional PICC placement	NR	NR	Tunneled: 32 (3–377); conventional: 33.5 (0–544)	Total catheter indwelling times: 6,972 days and 7,574 days with median durations of 15.5 days (range, 2–188 days) and 16.0 days (range, 2–134 days) in tPICC and cPICC groups
Lissauer et al. [21]	5.20%	Male sex, CCI 2, higher APACHE IV score, severity of illness, higher predicted ICU mortality, admission to the emergency surgery service, CPT 49002 was used as a surrogate for the open abdomen, admission to the SICU from another unit in the hospital for higher level of care, and readmission to the SICU during the same hospital stay following a previous SICU admission	NR	NR	NR	NR
Rhee et al. [22]	0.35 per 1,000 patient days	Hematologic malignancy	18.30%	NR	16±13.3	
Advani et al. [18]	2.01 per 1 000 catheter-days	Catheter duration	NR	NR	NR	1-60 days
Torre et al. [26]	3.9 per 1 000 catheter-days	More than one CVC at once, longer duration of CVC use	12.2%	NR	NR	NR
Wong et al. [23]	1.12 per 1,000 ICU CVC days	Double-lumen catheter insertion, CVC exposure > 7 days, and CVC insertion before 2011	NR	NR	NR	NR
Stevens et al. [19]	NR	APACHE II, multiple catheters, days in the ICU, multiple surgery, longer days in stepdown care, CCI, and DRG	CLABSI: 28.4% No CLABSI: 9%	CLABSI: 118,823 (172,555) USD; no CLABSI: 25,976 (44,270) USD	Pre-CLABSI length of stay: 24.0 days (30.0 days); post-CLABSI length of stay: 18.0 days (27.0 days); CLABSI: 43.0 (54.0); no CLABSI: 13.0 (18.0)	NR
Jeong et al. [32]	Phase 1: 3.7 per 1 000 catheter-days; phase 2: 2.1 per 1 000 catheter-days	Prolonged central line dwell days, use of a silicone catheter, surgical treatment, and less probiotic supplementation	NR	NR	CLABSI: 83.74±46.18; control: 64.77±42.73	NR
Malek et al. [27]	6 cases per 1,000 central line-days	Long ICU stay of 5 days or more, mechanical ventilation and the presence of heart failure long ICU stay of 5 days or more, mechanical ventilation and the presence of heart failure long ICU stay of 5 days or more, mechanical ventilation and the presence of heart failure long ICU stay of 5 days or more, mechanical ventilation and the presence of heart failure	16.8%	NR	CLABSI: ≥ 5 days (92.5%); no CLABSI: ≥5 days (7.5%)	NR
Ahn et al. [30]	3.7%	NR	36.3%	NR	NR	NR
Baier et al. [31]	18.2%; IR of 10.6 cases per 1,000 CVC days	CVC insertion for conditioning for stem cell transplantation, use of more than one CVC per case, leukocytopenia (≤1,000/μL), acute myeloid leukemia, carbapenem therapy, and pulmonary diseases	CLABSI: 7%; no CLABSI: 4%	8,810€ per case	CLABSI: 47 days; no CLABSI: 22 days	NR
Khieosanuk et al. [20]	3.2 per 1,000 catheter -days	Number of CVC lumen and place of catheter insertion	NR	NR	NR	NR

Seven of the studies reported on CLABSI-related mortality [31], two studies reported on associated hospital costs [31], and eight studies reported on the length of hospitalization [19,22,25,27,31,32]. With respect to predisposing factors, multilumen access catheters were identified as risk factors in three studies [20,23,24], use of more than one central venous catheter (CVC) per case in four studies [19,26,28,31], hematologic malignancy in three studies [22,24,31], catheterization duration in four studies [18,26,29,32], four surgical complexities in four studies [19,21,29,32], length of ICU stay in three studies [19,21,27], and parenteral nutrition in two studies [28,32].

Discussion

CLABSI is a matter of significant importance due to its profound impact on patient outcomes, healthcare costs, and the emergence of antibiotic resistance. The occurrence of CLABSI can lead to several adverse effects, such as prolonged hospital stays, increased morbidity and mortality rates, and escalated healthcare expenses [19]. Patients who develop CLABSI are at risk of developing sepsis, organ failure, and other serious complications, and may require additional treatment and care. By studying the risk factors, prevention strategies, and treatment options for CLABSI, researchers can identify ways to reduce the incidence and impact of this infection on patients. Furthermore, CLABSI is associated with significant healthcare costs, including the cost of additional treatment, longer hospital stays, and increased use of antibiotics. It is necessary to increase our understanding of the economic burden of CLABSI to help identify ways to reduce costs and improve the efficiency of healthcare delivery. Also, CLABSI is a significant driver of antibiotic resistance, as the use of antibiotics to treat these infections can lead to the development of resistant strains of bacteria [34]. By studying the epidemiology and microbiology of CLABSI, it is possible to arrive at novel approaches to reduce the use of antibiotics and prevent the development of antibiotic-resistant infections. Lafuente Cabrero et al. published a systematic review and meta-analysis in 2023 in which they synthesized and established the risk factors for CLABSI [35]. Their findings revealed that several factors increased the risk of developing CLABSI, including multilumen access catheters, the use of total parenteral nutrition, undergoing chemotherapy, being immunosuppressed, and prolonged duration of catheterization. Conversely, they found that monolumen devices were associated with a lower chance of causing this infection [35]. Belloni et al. published a systematic review on the occurrence rate and risk factors for long-term CLABSI in cancer patients in 2022 [36]. They noted a pooled occurrence rate of CLABSI of around 8% (95% CI: 4-14%). The main risk factors for long-term catheter-related infection in cancer patients were found to be the characteristics of the catheter device, management practices related to the catheter, administration of therapies, and individual clinical features of the patients [36]. Chopra et al. conducted a comprehensive review and analysis to examine the risk of CLABSI in adult patients with peripherally inserted central catheters (PICCs) compared to those with CVCs [37]. The meta-analysis of the studies demonstrated that PICCs were linked with a lower risk of CLABSI compared to CVCs, with a relative risk (RR) of 0.62 and a 95% CI of 0.40-0.94. Subgroup analysis revealed that this risk reduction was most pronounced in ambulatory patients (RR [95% CI]: 0.22 [0.18-0.27]) compared to inpatients who received PICCs (RR [95% CI]: 0.73 [0.54-0.98]) [36]. The incidence rate of PICC-related CLABSI was found to be similar to that of CLABSI from CVCs (incidence rate ratio [95% CI]: 0.91 [0.46-1.79]). The authors concluded that while PICCs were associated with a lower susceptibility to CLABSI than CVCs in ambulatory patients, the likelihood of CLABSI with PICCs, as with CVCs, remained higher for admitted patients [37].

Included Publications Reporting on the Adult Population

Concerning studies included in our systematic review, Herc et al. studied factors linked with the use of PICC and CLABSI incidence to create a risk model for estimating individual risk of PICC-associated CLABSI before catheterization [24]. Significant predisposing factors linked to PICC-CLABSI included hematologic malignancy, previous CLABSI within three months of PICC insertion, use of a multilumen access PICC, ongoing chemotherapy for solid cancers, administration of total parenteral nutrition through the PICC, and concurrent use of another CVC at the time of PICC insertion [24]. In their study, Hernández-Aceituno et al. compared CLABSI incidence pre- and post-implementation of a set of infection control measures [28]. They also attempted to detect risk factors for CLABSI following the implementation of insertion bundle, which consisted of the subclavian vein as access of choice, disinfection with alcoholic 2% chlorhexidine, central-line full body drapes, sterile ultrasound probe-cable covers, and insertion checklist. They found that prior to the implementation of these measures, the cumulative incidence (IC) of CLABSI was 5.05% and the incidence density rate (IR) was 5.17%. Following the implementation of new measures, there was a reduction of 54.8% in IC (p = 0.072) and of 56% in IR (p = 0.068). In multivariable analyses, replacement of CVC was associated with a higher risk of CLABSI (OR: 11.01; 95% CI: 2.03-59.60; p = 0.005), as well as two or more catheterizations (OR: 10.05; 95% CI: 1.77-57.16; p = 0.009), and parenteral nutrition (OR: 23.37; 95% CI: 4.37-124.91; p < 0.001). They observed a lower rate of CLABSI following the adoption of new measures. They also concluded that replacing CVCs, using more than one catheter, and providing nutrition parenterally increased the risk of CLABSI after the new measures were implemented [28]. Kim et al. assessed the impact of subcutaneous tunnelling on PICC insertion with respect to CLABSI. CLABSI was observed to be significantly less common after tunnelling (8/6,972 catheter days) than after conventional PICC placement (28/7,574 catheter days; adjusted hazard ratio = 0.328; 95% CI: 0.149-0.721) [25]. Other predisposing factors such as age, sex, comorbid conditions, PICC duration, veins, hospitalization, and ICU stay showed no significant correlations with CLABSI. They concluded that, compared with the traditional approach, a subcutaneous tunnelling approach for PICC insertion significantly lowered the occurrence of CLABSI [25]. In a study conducted by Lissauer et al., the authors examined the risk factors linked with CLABSI. Their findings revealed that patients who were critically ill upon their admission to the intensive care unit (ICU) exhibited a higher Acute Physiology and Chronic Health Evaluation (APACHE IV) score compared to less critically ill patients (85.2 ± 21.9 vs. 65.6 ± 23.2; p < 0.01). Additionally, the study identified that these critically ill patients had a greater likelihood of being admitted to the emergency surgery service (OR: 1.92; 95% CI: 1.02-3.61) and showed a significant association with the reopening of a recent laparotomy (OR: 2.08; 95% CI: 1.10-3.94). They inferred that in settings where best practices are followed, CLABSI patients show distinctive clinical features as compared to non-CLABSI patients, which may point to patient populations that require enhanced preventive approaches [21]. Rhee et al. d examined various factors and proposed that individuals undergoing dialytic therapy demonstrated a higher prevalence of CLABSI [22]. They conducted the study over a period of two years and found that the mean duration of hospital stay before CLABSI occurrence was 16 ± 13.3 days, which was nearly three times longer than the non-ICU length of stay for the entire hospital population. Among the patients, only 11 (10.6%) received dialysis within 48 hours of developing CLABSI. However, 67% of the patients had a hematologic malignancy, and among those admitted with a malignant hematologic diagnosis, 91.8% were neutropenic at the time of CLABSI. The most commonly isolated pathogen was Enterococcus spp., and half of all CVCs in place were peripherally inserted. The overall mortality rate was 18.3%, while among dialysis patients, it was 27.3%. The researchers reached the conclusion that the presence of underlying neutropenia, hematologic cancer, and the use of PICC lines were notably common among the patients affected by CLABSI [22]. Wong et al. explored the risk-adjusted association between ICU-acquired CLABSI and in-hospital mortality [23]. The overall rate of CLABSI in the ICU was 1.12 per 1,000 ICU days with a CVC. Several significant independent risk factors were identified for CLABSI acquired in the ICU, including the insertion of a double-lumen catheter (OR: 2.59; 95% CI: 1.16-5.77), CVC insertion prior to 2011 (OR: 2.20; 95% CI: 1.22-3.97), and CVC exposure for more than seven days (OR: 2.07; 95% CI: 1.06-4.04). Although ICU-acquired CLABSI was initially associated with higher in-hospital mortality, this effect was reduced after adjusting for the likelihood of acquiring CLABSI (OR: 1.20; 95% CI: 0.54-2.68). The researchers concluded that a higher likelihood of ICU-acquired CLABSI was associated with increased in-hospital mortality, but the infection itself was not directly responsible. They inferred that the requirement for prolonged specialized central venous access played a significant role in the development of ICU-acquired CLABSI, which could potentially contribute to mortality as an indicator of ongoing organ dysfunction [23]. Stevens et al. studied the link between CLABSI and increased hospital costs and mortality risk [19]. After adjusting for the severity of illness and other healthcare-associated infections, it was found that CLABSI was associated with a 2.27-fold increase in the risk of mortality (95% CI: 1.15-4.46). In general, CLABSI was significantly associated with higher adjusted in-hospital mortality rates as well as increased total and variable costs compared to patients who did not have CLABSI [19]. Malek et al. measured the incidence, predisposing factors, and most frequent causative pathogens of CLABSI at a private hospital [27]. The overall IR of CLABSI was six cases per 1,000 central line days. The central line utility rate was 0.94 per 1,000 patient-days. The rate of central line utilization was 0.94 per 1,000 patient-days. During the study period, the mortality rate in cases with CLABSI was 16.8% (95% CI: 13.6-20.4%). Univariate analysis identified several predisposing factors for CLABSI, including comorbid conditions such as heart failure, APACHE II scores of >15, ICU stays of five days or more, duration of CVC placement, subclavian placement of CVCs, and mechanical ventilation. Logistic regression analysis further revealed that a mechanical ventilation, prolonged ICU stay of five days or more, and the presence of cardiac failure were the only significant predictors. Gram-negative bacteria, particularly Enterobacter (36.8%; 95% CI: 16.3-61.6%) and Pseudomonas aeruginosa (21.1%; 95% CI: 16.0-45.5%), were the most commonly identified pathogens in cases of CLABSI [27]. Ahn et al. studied the incidence and clinical impact of CLABSI in adult patients who underwent central line insertion in the emergency department (ED) [30]. CLABSI was defined if the same pathogens were identified at peripheral and catheter tips or the differential time to positivity was >2 hours. Those with CLABSI had a higher incidence of subclavian vein insertion and retry rates. *Staphylococcus epidermidis* was the most common pathogen, followed by *Staphylococcus aureus*, *Enterococcus faecium*, and *Escherichia coli*. Using multivariate analysis, they found that CLABSI development was an independent risk factor for in-hospital mortality (adjusted OR: 1.93; 95%, CI: 1.19-3.14; p < 0.01). They concluded that CLABSI after central line placement in ED is common and linked with poor outcomes [30]. Baier et al. conducted a study to examine the occurrence, risk factors, and healthcare costs associated with CLABSI in patients with hematologic and oncologic conditions [31]. They identified several independent risk factors for CLABSI, including the use of multiple CVCs per case, CVC insertion for conditioning prior to stem cell transplantation, acute myeloid leukemia, leukocytopenia (≤1,000/μL), carbapenem therapy, and lung diseases. The study also found that the occurrence of CLABSI was associated with hospital costs of 8,810€ per case, highlighting the significant impact of CLABSI on overall healthcare costs [31].

Rabelo et al. published a systematic review and meta-analysis on risk factors for CLABSI during pediatric cancer therapy in 2023 [38]. They noted that diagnosis of hematologic neoplasm, the intensity of treatment, blood transfusion in the four to seven days before the infection, type of long-term catheters (tunnelled externalized catheters, double lumen, greater diameter), inpatient treatment, and a longer period of hospitalization were the most consistent risk factors. The meta-analysis revealed that neutropenia at the time of catheter placement was not a risk factor for CLABSI, although there was a high heterogeneity between studies. Staphylococcus epidermidis was the most common pathogen reported [38].

Included Publications Reporting on the Pediatric Population

DiPietro et al. explored the risk factors for CLABSI in pediatric cardiac critical care units [29]. In surgical hospitalizations, a CVC was used in 88% of cases, whereas in medical hospitalizations, the usage rate was 35%. The internal jugular vein was the most common site for CVC placement, accounting for 46% of cases. The median duration of CVC placement was four days. Among all hospitalizations, there were 248 cases (2% overall, 1.8% medical, and 2.1% surgical) with at least one central line-associated thrombosis, resulting in a total of 271 thrombosis. Thrombosis was typically diagnosed around seven days after catheter placement. Furthermore, there were 127 hospitalizations (1% overall, 1.4% medical, and 1% surgical) with at least one CLABSI, resulting in a total of 136 infections. There was no significant association found between the type or site of the catheter and the occurrence of CLABSI. It was diagnosed at a median of 19 days after catheterization. In this study, significant predisposing factors for central line-associated thrombosis and CLABSI included younger age, the duration of catheterization, and the higher surgical complexity. Among these factors, the total number of CVC line days was the only modifiable risk factor identified [29]. Advani et al. studied risk factors for PICC-CLABSI in a hospitalized pediatric population, particularly children receiving non-ICU care [18]. A total of 116 CLABSIs occurred over 44,972 catheter-days, resulting in an incidence rate of 2.58 cases per 1,000 catheter-days (95% CI: 2.07-3.00 cases per 1,000 catheter-days). Independent predictors of CLABSI in the entire population included a PICC placement duration of more than 21 days (incidence rate ratio [IRR]: 1.53; 95% CI: 1.05-2.26), the indication for insertion being parenteral nutrition (IRR: 2.24; 95% CI: 1.31-3.84), prior PICC-associated CLABSI (IRR: 2.48; 95% CI: 1.18-5.25), underlying metabolic conditions (IRR: 2.07; 95% CI: 1.14-3.74), and exposure to the pediatric intensive care unit (PICU) during hospitalization (IRR: 1.80; 95% CI: 1.18-2.75). Risk factors for CLABSI in children without PICU exposure were found to be younger age, underlying cancer and metabolic disorders, PICCs inserted in the lower extremity, and a previous CLABSI associated with a PICC. They inferred that prolonged catheterization, PICU exposure, use of PICC as a parenteral route for nutrition administration increased susceptibility for PICC-CLABSI in pediatric inpatients [18]. Torre et al. studied risk factors for PICU-linked CLABSI [26]. The rate of CLABSI was 3.9 cases per 1,000 CVC days. The incidence rate varied across hospitals, ranging from 1.6 to 6.6 cases per 1,000 catheter-days. The overall mortality rate was 11.1%, with CLABSI cases having a mortality rate of 12.9% and non-CLABSI cases having a mortality rate of 10.7%. After conducting a multivariate analysis, two independent risk factors for CLABSI were identified: longer duration of CVC use (OR: 1.07; 95% CI: 1.00-1.14; p = 0.019) and the use of multiple CVCs simultaneously (OR: 2.59; 95% CI: 1.17-5.73; p = 0.048) [26]. In a study by Jeong et al., the impact of modifying risk factors to control CLABSI among high-risk infants in a tertiary neonatal intensive care unit was examined [32]. The study identified several risk factors associated with CLABSI, including prolonged central line dwell days (adjusted HR: 1.028; 95% CI, 1.011-1.045; p=0.001), usage of a silicone catheter (adjusted HR: 5.895; 95% CI: 1.893-18.355; p=0.002), undergoing surgery (adjusted HR: 3.793; 95% CI: 1.467-9.805; p=0.006), and lower probiotic supplementation (adjusted HR: 0.254; 95% CI: 0.068-0.949; p=0.042). By implementing a quality improvement initiative targeting these risk factors, the average incidence rate of CLABSI per 1,000 catheter-days decreased significantly from 6.6 to 3.1 (p=0.004) [32]. Khieosanuk et al. studied the incidence and risk factors of CLABSI among neonates (aged < 1 month) and children (aged ≥ 1 month) admitted to a tertiary care university hospital [20]. An overall CLABSI incidence rate was 3.2 per 1,000 catheter-days. Among neonates, 12 (3%) CLABSI episodes occurred, corresponding with IR of 3.1 (95% CI: 1.8-5.5) per 1,000 catheter-days. For children, 18 (3%) CLABSI events were observed, accounting for IR of 3.3 (95% CI: 2.1-5.3) per 1,000 catheter-day. Out of a total of 131 deaths, three were CLABSI-related mortality (one neonate; two children). A number of CVC lumen and place of catheter insertion were a significant risk factor among our neonates and children, respectively. CLABSI lengthened hospitalization and elevated hospital costs [20].

Strength and limitation

Our study aimed to provide a comprehensive review of existing data on the topic. It is important to note that there was considerable heterogeneity among the included studies in terms of their research endpoints, which can be seen as a limitation. This heterogeneity may be attributed to the use of observational data, as randomization is not feasible in such studies. The selection of controls in observational studies can introduce selection bias. Additionally, the quality of the included studies varied, posing another limitation. To establish more conclusive evidence, further studies with consistent endpoints are needed. It is worth mentioning that not all of the included studies provided information on certain aspects such as CLABSI-associated mortality, catheter days, length of hospitalization, and hospital costs.

## Conclusions

Understanding predisposing factors is a vital step to reduce morbidity and mortality related to CLABSI. It is important to make individualized decisions regarding the insertion of venous devices based on the evaluation of risk factors to prevent the development of CLABSI, as this complication can have severe clinical consequences and result in significant healthcare costs. There is a need for policy and procedural oversight regarding catheter insertion and maintenance in order to improve patient outcomes. Future well-designed studies focusing on pathogenesis and standardized insertion practices with homogeneous patient samples are necessary to enhance the quality of the findings. The healthcare costs associated with CLABSI impose a substantial burden on hospitals. Although a decrease in CLABSI rates can help alleviate some of these expenses, not all costs can be offset. Future research should explore expenditures while considering the timing of infection in both ICU and non-ICU patients. The clinical and financial impact of CLABSI underscores the importance of strictly adhering to recommended infection control practices. By reducing CLABSI, there will be a significant reduction in morbidity, mortality, and healthcare expenditure.
